# Clinical microbiology in developing countries.

**DOI:** 10.3201/eid0702.010232

**Published:** 2001

**Authors:** L K Archibald, L B Reller

**Affiliations:** Centers for Disease Control and Prevention, Atlanta, Georgia 30333, USA. lca6@cdc.gov

## Abstract

We review the problem of limited microbiology resources in developing countries. We then demonstrate the feasibility of a cohort-based approach to integrate microbiology, epidemiology, and clinical medicine to survey emerging infections in these countries.


### Clinical Microbiology in Developing Countries

**Lennox K. Archibald* and L. Barth Reller†**
*Centers for Disease Control and Prevention, Atlanta, Georgia, USA, and
†Duke University Medical Center, Durham, North Carolina, USA

We review the problem of limited microbiology resources in developing countries. We then demonstrate the feasibility of a cohort-based approach to integrate microbiology, epidemiology, and clinical medicine to survey emerging infections in these countries.

### Microbiology Resources in Developing Countries

In industrialized countries, it is the best of times for the microbiologic diagnosis and treatment of infections. In some developing countries, progress is also apparent. Ministries of health are building hospital intensive care units (ICUs), with sophisticated medical devices, procedures, and interventions. Increasing numbers of infants and adults are being admitted to, and benefiting from, these units. More patients with conditions such as chronic renal failure or hematologic disorders are being treated in specialized units. The Internet has made physicians generally more knowledgeable than before.

Nevertheless, it is the worst of times for hospitals in other developing countries, where infectious diseases remain the leading cause of death (1). Many sentinel hospitals have less than basic microbiology laboratory facilities; there is no end in sight to the HIV epidemic, and the prevalence rate of tuberculosis (TB) is increasing in parallel with it; hospital infections, especially surgical site infections, have become important causes of illness and death in certain hospitals in sub-Saharan Africa (unpublished data); and invasive medical devices and procedures are increasingly being introduced into ICUs and operating theaters without the necessary infection control procedures. In some developing countries, some institutions have all the needed microbiologic resources, while others have none; some hospital laboratories have instruments and reagents yet have no technical staff to use them; others may be able to amplify genomes yet cannot report the results of a simple Gram stain in a timely manner. For all these reasons, the causes of many infections among inpatients in Africa, Southeast Asia, the Indian subcontinent, and parts of the Americas remain largely unknown or uncharacterized.

In sub-Saharan Africa and Southeast Asia, antimicrobial-drug resistance is being increasingly recognized in pathogens that commonly cause infections in health-care settings, rendering available antimicrobial agents ineffective and further shortening the list of already scarce effective agents (2). Thus, to diagnose and treat infections appropriately and to fully characterize emerging infections in developing countries, enhanced clinical microbiology services are a priority. The clinical microbiology laboratory in developing countries should be patient directed and guided by clinical reality and not by high technology or outside interests.

Address for correspondence: Lennox K. Archibald, Division of Healthcare Quality Promotion, Centers for Disease Control and Prevention, Mailstop A35,1600 Clifton Road, Atlanta, GA 30333, USA; fax: 404-639-2647; e-mail: lca6@cdc.gov

Two other factors have had a marked effect on the role of clinical microbiology in developing countries, the HIV and TB epidemics. Most (95% of the global total) people with HIV infection live in the developing world (3,4). In almost 6 million of the 34 million adults and children with HIV or AIDS, HIV infection was diagnosed during 1999 (4); 3.8 million cases occurred in sub-Saharan Africa and 1.3 million in South and Southeast Asia. Of the approximately 40 million TB cases globally, 73% are projected to have occurred in Southeast Asia and sub-Saharan Africa (5). TB, which accounts for almost one third of the AIDS deaths worldwide, and other opportunistic bacterial, fungal, and protozoal infections are leading causes of death among HIV-infected patients (3-5). Thus, HIV infection, TB, and HIV-related opportunistic infections have overwhelmed existing resources in hospital microbiology laboratories in most developing nations.

At the Centers for Disease Control and Prevention (CDC), a main objective of the strategy for preventing and controlling emerging infectious diseases in developing countries is establishing more effective international surveillance networks (6). In the industrialized world, infection control relies on results from individual patient-directed diagnostic microbiology laboratory tests. However, basic clinical microbiology has not been recognized as a priority by donor or governmental agencies in industrialized countries or by the developing countries themselves. The problem often has been compounded by lack of trained laboratory personnel or prohibitive costs associated with maintaining a laboratory. Where resources are available, they may be used inappropriately (e.g., nonessential stool, urine, or sputum cultures; antimicrobial susceptibility testing of microorganisms without quality assurance; or complete laboratory characterization and antimicrobial susceptibility testing of bacterial isolates that are not clinically relevant).

Prohibitive costs and doubtful cost-effectiveness of specific tests are commonly cited as reasons for the unavailability of microbiology tests. The first steps in achieving cost-effective use of resources include assessing whether or not a test has sufficient diagnostic value to be used and establishing criteria to limit processing to those organisms most likely to be clinically relevant (7). The concept of clinical value encompasses several issues (8): Why was the test requested? Will the result help or alter patient management? Would a simpler test do? Will the use of a test increase knowledge? Can we do without it? Is the test of public health or clinical importance? For example, hospitals in developing countries still routinely obtain and process anaerobic blood cultures, despite that a positive anaerobic blood culture often reflects an underlying anaerobic infection (e.g., intraabdominal sepsis or female genital tract infection) that already is clinically apparent or discernible (9,10). The counter argument is that while such data may reflect reality for a microbiology issue in industrialized nations, they may not be applicable for developing settings—all the more reason for important questions about diagnostic clinical microbiology in developing countries to be addressed through evidence-based clinical studies.

The importance of integrating epidemiology and microbiology is exemplified by studies that ascertained the usefulness of expensive HIV confirmation tests in developing countries. In industrialized countries, confirmation of HIV serologic tests with the Western blot molecular technique is standard practice. In developing countries, the Western blot often is not used because of its complexity and high cost. A study conducted in Thailand with epidemiologic, clinical, and microbiologic components has shown that the use of two enzyme-linked immunosorbent assays (ELISAs) to confirm the presence of HIV antibodies produces results comparable with those of the Western blot (11). This approach to confirming HIV status was used effectively in Tanzania (12), Thailand (13), and Malawi (14). Thus, in a country with a high prevalence rate of HIV infection, limited financial resources, and inadequate laboratory infrastructure, Western blot analysis for confirmation of HIV infection is neither mandatory nor necessary.

Medical services in industrialized nations rely on results from individual, patient-directed, diagnostic microbiology laboratory tests ordered by clinicians. This system appears effective for industrialized settings and is generally sustainable. Not surprisingly, diagnostic microbiology services in some developing countries have been modeled on these practices in industrialized countries. However, such routine laboratory testing may be impossible in developing settings because of lack of microbiology services, or, where these services are available, tests may be unreliable if performed improperly or without adequate quality control. Further, the tests may well be inappropriate, irrelevant, or redundant. For example, antimicrobial susceptibility testing without quality controls may lead to invalid or distorted data that give rise to bias and inaccuracy in reports being used for clinical and public health decision making.

### Hospital Cohort-Based Studies

During the past few years, the Hospital Infections Program at CDC and the Clinical Microbiology Laboratory at Duke University Medical Center participated in hospital cohort-based microbiologic surveys. These surveys are conducted with a cohort of patients who meet simple, objective entry criteria or case definitions (e.g., fever, diarrhea, cellulitis, or specific syndromes). Detailed clinical and epidemiologic data are collected for later analyses, and cultures with a high positive predictive value for infection (e.g., blood, cerebrospinal fluid, other sterile sites, or stool for enteric pathogens) are obtained. The emphasis is on performing quality-controlled laboratory testing for a finite period rather than long-term, routine diagnostic testing. These surveys have been conducted in selected hospitals or laboratories that provide a natural gathering point to sample patients meeting these entry criteria. A cohort-based study acting as a surveillance "probe" for a finite period may be more effective than individual patient-directed laboratory testing in providing useful clinical and public health information, in determining the true incidence and prevalence rates of emerging pathogens and antimicrobial-drug resistance, and in yielding clinical predictors for various infections in defined patient cohorts. In addition, cohort-based studies provide the opportunity to establish diagnostic capability in basic clinical microbiology in sentinel hospitals or laboratories and promote surveillance activities in regions where critical public health infrastructure has been neglected.

### Cohort Studies of Bloodstream Infection

To test this approach to clinical microbiology, CDC and Duke conducted cohort-based studies of bloodstream infections among inpatients in sub-Saharan Africa and Southeast Asia (12-15). Fever was chosen as the initial case definition because it may be attributed to HIV infection, diarrhea, pneumonia, TB, or, in sub-Saharan Africa, malaria. Blood cultures were obtained because of their high positive predictive value for presence of bloodstream infections in febrile patients.

In Thailand about half of consecutive febrile adults admitted to a sentinel teaching hospital for infectious diseases had bloodstream infections (13); in a similar patient cohort in a Malawi teaching hospital, approximately one quarter of patients had a bloodstream infection (14). In both countries, *Mycobacterium tuberculosis*, *Streptococcus pneumoniae*, and *Salmonella* spp. were the predominant causes of bloodstream infections in these patients. Data from these studies also included clinical predictors for bloodstream infections and antimicrobial susceptibility profiles of clinically important isolates (including *M. tuberculosis* isolates). Both the predictors and susceptibility profiles were potentially useful for developing algorithms for empiric treatment of febrile inpatients and for helping clinicians decide which patients would most benefit from limited blood culture services, where these were available. Through cohort-based studies in Malawi during the dry and wet seasons, we demonstrated seasonal variation in bloodstream infection: *S. pneumoniae* and *M. tuberculosis* were the predominant bloodstream pathogens during the dry season, whereas *Salmonella* spp. were the predominant bacteria isolated during the wet season (16). We also documented that malaria was overdiagnosed in both the wet and dry seasons in Malawi and that empiric therapeutic decisions had to reflect these realities (16).

Cohort-based studies in Thailand and Malawi demonstrated the occurrence of occult mycobacteremia (15): 42% of patients with *M. tuberculosis* bloodstream infections had neither symptoms nor signs of pulmonary TB. These results highlighted the importance of maintaining a low threshold of suspicion for active TB; the need for strengthening each hospital's microbiology capabilities to examine and report on sputum smears for acid-fast bacilli; and the potential for intrahospital TB transmission from seemingly noninfectious patients.

The public health implications of the cohort-based approach are enormous. Conducting similar studies in other countries would improve microbiology services by encouraging appropriate use of limited resources in sentinel hospital laboratories and focusing on clinically relevant problems (e.g., bloodstream infections, meningitis, pneumonia, febrile diarrhea, and surgical wounds). Moreover, laboratory personnel would benefit from training to conduct quality-controlled tests, such as antimicrobial-drug susceptibility testing. Prevalence rates of common infections, HIV infection, or resistance of common hospital pathogens to available antimicrobial agents would be available for clinical and public health decision making. Updated lists of probable diagnoses, clinical predictors for specific infections, and development of clinical algorithms and antimicrobial-drug susceptibility profiles based on these objective data would enhance patient care through rational diagnosis and prescribing policies.

Although it may not be economically feasible to obtain cultures for all patients who might benefit from microbiologic tests in developing countries, cohort-based studies could be applied to establish the causes and clinical predictors for these infections and thereby facilitate directed rather than blind empiric therapy.

Hospital laboratories in developing countries need to establish screening and rejection criteria for specimens submitted for culture. Laboratory directors need to address certain questions: Will the results alter patient management? What is the public health importance? What is the relative yield of a Gram-stained smear versus a complete culture?

Data from cohort-based studies in one region or country are not suitable for direct extrapolation to other regions or countries. Rather, regional, season-specific surveillance studies can be tools for optimizing patient care where routine laboratory testing is not available. The task remains to define the role of new and emerging pathogens in various patient populations at hospitals in developing countries.

### The Role of Sentinel Hospitals

During the past 5 years, our cohort-based approach to collaborative global endeavors in health-care epidemiology has included identifying sentinel hospitals and then enhancing their clinical microbiology laboratory capacity by infection control assessments and interventions. In developing countries, where limited resources and infrastructure may preclude comprehensive medical, surgical, and laboratory services for every region or province, centralization of available resources in a few selected centers is one way of optimizing resources. This paradigm is evident in many countries in Southeast Asia, Africa, Latin America, and the Caribbean, where a few institutions have evolved into sentinel centers of paramount importance for providing such services.

Sentinel hospitals tend to be large institutions (usually >500 beds) that are the main teaching centers for medicine, surgery, nursing, and laboratory science; they commonly house specialized ICUs, surgery, hemodialysis, or invasive medical procedures; they have problems with hospital infections and antimicrobial-drug resistance; they are associated with microbiology laboratories that are often reference centers with the ability and capacity to conduct various microbiologic tests using scrupulous, quality-controlled methods; and they usually are government affiliated and have very close links with the respective ministry of health. The last attribute is important since governmental agencies from industrialized countries (e.g., World Health Organization [WHO], United States Agency for International Development, and the Department for International Development) generally prefer to maintain collaborative endeavors with sentinel centers for reasons including adequate infrastructure, trained personnel, and access to the ministry of health.

A high priority for future global consortiums of epidemiology and biomedical research centers will be to initiate or build upon existing systems in sentinel hospitals in developing countries for the international monitoring and reporting of antimicrobial susceptibility data. Two systems that offer a foundation of international linkages are CDC's International Nosocomial Surveillance Program for Emerging Antimicrobial Resistance and the WHO Antimicrobial Resistance Monitoring Program. The international and national objectives of these programs depend on conducting proper, quality-controlled, antimicrobial susceptibility testing and promoting the use of resistance data to guide antimicrobial therapy. These results, when integrated with clinical and epidemiologic data on opportunistic and hospital infections, may lead to substantial improvement in patient outcomes.

Dr. Archibald is a medical epidemiologist in the Division of Healthcare Quality Promotion (formerly the Hospital Infections Program), CDC. He is a member of the Royal College of Physicians of the United Kingdom and is trained in internal and tropical medicine, infectious diseases, and clinical microbiology. His research interests include the impact of cost-effective clinical microbiology services on patient outcome.

Dr. Reller is professor of medicine (infectious diseases) and pathology (medical microbiology) and director of clinical microbiology at Duke University Medical Center. His research interests include the diagnosis and epidemiology of bloodstream and mycobacterial infections.

### References

1. Hinman AR. Global progress in infectious disease control. Vaccine 1998;16:1116-21.
2. Hart CA, Kariuki S. Antimicrobial resistance in developing countries. BMJ 1998;317:647-50.
3. Grant AD, De Cock KM. The growing challenge of HIV/AIDS in developing countries. Br Med Bull 1998;54:369-81.
4. World Health Organization. AIDS epidemic update: December 1999. Geneva: Joint United Nations Programme on HIV/AIDS (UNAIDS); 1999.
5. World Health Organization. Global tuberculosis control. Geneva: WHO; 2000.
6. Centers for Disease Control and Prevention. Addressing emerging infectious disease threats: a prevention strategy for the United States. Atlanta: CDC; 1994.
7. Robinson A. Rationale for cost-effective laboratory medicine. Clin Microbiol Rev 1994;7:185-99.
8. Spencely M, Parker MJ, Dewar RAD, Miller DL. The clinical value of microbiological investigations. J Infect 1979; 1:23-6.
9. Ortiz E, Sande MA. Routine use of anaerobic blood cultures: are they still indicated? Am J Med 2000;108:445-7.
10. Bartlett JG, Dick J. The controversy regarding routine anaerobic blood cultures. Am J Med 2000;108:505-6.
11. Ittiravivongs A, Likanonsakul S, Mastro TD, Tansuphasawadikul S, Young N, Naiwatanakul T, et al. Evaluation of a confirmatory HIV testing strategy in Thailand not using western blot. J Acquir Immune Defic Syndr 1996;13:296-7.
12. Archibald LK, den Dulk MO, Pallangyo KJ, Reller LB. Fatal *Mycobacterium tuberculosis* bloodstream infections in febrile hospitalized adults in Dar es Salaam, Tanzania. Clin Infect Dis 1998;26:290-6.

13. Archibald LK, McDonald LC, Rheanpumikankit S, Tansuphaswadikul S, Chaovanich A, Eampokalap B, et al. Fever and human immunodeficiency virus infection as sentinels for emerging mycobacterial and fungal bloodstream infections in hospitalized patients 15 years old, Bangkok. J Infect Dis 1999;180:87-92.
14. Archibald LK, McDonald LC, Nwanyanwu O, Kazembe P, Dobbie H, Tokars J, et al. A hospital-based prevalence survey of bloodstream infections in febrile patients in Malawi: implications for diagnosis and therapy. J Infect Dis 2000;181:1414-20.
15. McDonald LC, Archibald LK, Rheanpumikankit S, Tansuphaswadikul S, Eampokalap B, Nwanyanwu O, et al. Unrecognised *Mycobacterium tuberculosis* bacteraemia among hospital inpatients in developing countries. Lancet 1999;354:1159-63.
16. Bell M, Archibald LK, Nwanyanwu O, Kazembe P, Dobbie H, Reller LB, et al. Seasonal variation in the etiology of bloodstream infections in a febrile inpatient population in a developing country. Int J Infect Dis 2001 (in press).
